# Real-world experiences and treatment patterns among congenital heart disease patients with associated pulmonary vascular disease: Results from a real-world survey in the United States

**DOI:** 10.1016/j.jhlto.2025.100326

**Published:** 2025-06-20

**Authors:** Jordan D. Awerbach, Carly J. Paoli, Megan Scott, Gurinderpal Doad, Julia Harley, Daniel Graham, Mark Small, Sumeet Panjabi, Leigh C. Reardon

**Affiliations:** a Center for Heart Care, Phoenix Children’s, Phoenix, AZ; b Departments of Internal Medicine and Child Health, University of Arizona College of Medicine-Phoenix, Phoenix, AZ; c Actelion Pharmaceuticals US Inc, San Francisco, CA; d Adelphi Real World, Bollington, United Kingdom; e Ahmanson/UCLA Adult Congenital Heart Disease Center and UCLA Mattel Children’s Hospital, University of California Los Angeles, Los Angeles, CA

**Keywords:** Pulmonary arterial hypertension, Congenital heart disease, Pulmonary vascular disease, Fontan circulation, Treatment patterns, Real-world evidence

## Abstract

**Background:**

Limited data exist on the use of drugs in patients with pulmonary arterial hypertension associated with congenital heart disease (PAH-CHD). Therefore, we evaluated their real-world patient journey, including symptomatology, diagnostic pathway, treatment patterns, and guideline adherence.

**Methods:**

Data were drawn from the Adelphi Real World pulmonary hypertension congenital heart disease Disease Specific Programme (DSP), a cross-sectional survey of clinicians and patients with PAH-CHD or Fontan circulation associated with elevated pulmonary vascular resistance (PVR). Data were collected in the United States from November 2021 to May 2022. Analyses were descriptive.

**Results:**

Overall, 51 clinicians reported data for 191 patients with PAH-CHD or Fontan circulation associated with elevated PVR. Fifty-eight patients voluntarily provided data. Overall, 10.5% of patients had a gap of ≥1 year in their disease management. Mean (standard deviation, SD) time from pulmonary hypertension symptom onset to diagnosis/confirmation was 1.7 (2.2) years. Overall, 75.0% of patients underwent a right heart catheterization (RHC) at diagnosis/confirmation. Clinicians reported that 75.9% of patients were prescribed treatment for their PAH-CHD or elevated PVR. Pulmonary hypertension specific therapy was prescribed as combination therapy for 47.6% of patients prescribed pulmonary hypertension specific treatment.

**Conclusions:**

Patients experienced delays to diagnosis and gaps in congenital heart disease management. We observed low utilization of RHC and combination therapy. Key unmet needs in this population include more frequent testing to shorten time-to-diagnosis and proactive management with initial combination therapy.

## Background

Pulmonary arterial hypertension (PAH) is a progressive disease associated with high morbidity and mortality.^1^ PAH is commonly associated with congenital heart disease (CHD);^2^ population-based studies suggest 6%-28% of adults with CHD will develop PAH,^3^, ^4^ while PAH has a CHD etiology in 19.5% of cases.^5^ PAH-CHD is a lifelong, progressive disease,^2^ associated with a two-fold increase in all-cause mortality and a three-fold increase in cardiac morbidity compared to that in CHD patients without PAH.^6^ PAH is associated with increased healthcare resource utilization and intensive care unit admissions.^6^

The 2023 American Heart Association scientific statement on pulmonary hypertension (PH) in CHD describes four clinical and hemodynamic PAH-CHD profiles, 1) Eisenmenger syndrome, 2) unrepaired moderate to large defects with mildly to moderately elevated pulmonary vascular resistance (PVR) with predominantly left-to-right shunt, 3) PAH with coincidental CHD, 4) persistent or recurrent PAH after surgical repair of CHD.^7^ The development of progressive pulmonary vascular disease is seen in the palliated single ventricle population and is a major contributor to failing Glenn and Fontan physiology.^8^ These patients do not fulfill standard criteria for PH but demonstrate an increased transpulmonary gradient and PVR.^2^, ^8^, ^9^ Elevated PVR in Fontan circulation is increasingly appreciated, although not fully addressed in European PH diagnosis and treatment guidelines.^10^

The PAH-CHD population is difficult to treat because of the heterogeneity and varying complexity of cardiac lesions.^11^, ^12^ The primary management strategy for most patients with PAH-CHD is medical therapy using a proactive disease-targeting therapy approach, although surgical palliation or shunt defect closure may be considered in certain cases.^2^, ^13^ Evidence suggests patients have improved outcomes when targeting foundational pathways (nitric oxide, endothelin, and prostacyclin) in PAH individually and in combination, clinicians have multiple options for daily (oral, inhaled) and continuous (intravenous, subcutaneous) therapy administration.^11^ Recent evidence suggests Fontan patients with elevated PVR may also benefit from PH-specific therapy, but more data are required to confirm early findings.^2^ An alternative treatment strategy involving initial treatment with PH-specific drugs to enable repair of the cardiac defect is known as “treat-to-close,” although larger data sets with longer follow-up are needed to define patients likely to benefit most from this option.^7^, ^14^

Few real-world studies specifically looking at PAH-CHD have been performed. Thus, we know little about the typical journey and experience of a patient with PAH-CHD, including initial diagnosis, testing, symptomatology, and treatment in real clinical practice.^15^, ^16^, ^17^, ^18^, ^19^, ^20^ This study aimed to elucidate unmet needs in this population and evaluate the real-world journey for these patients, with additional focus on management practices in the Fontan circulation associated with the elevated PVR patient population, where evidence is limited.

## Materials and methods

Data were drawn from the Adelphi Real World PH-CHD Disease Specific Programme (DSP), a cross-sectional survey, with elements of retrospective data collection, of clinicians and their patients with PAH-CHD or Fontan associated with elevated PVR. Data were collected in the United States (US) between November 2021 and May 2022. A complete description of the DSP methodology has been previously published and validated.^21^, ^22^, ^23^, ^24^

### Survey population

Clinicians (adult cardiologists, pediatric cardiologists, and adult pulmonologists) were eligible for inclusion if they were personally responsible for the management of patients with PAH-CHD or patients with Fontan circulation associated with elevated PVR (referred to as “Fontan patients”). Clinicians were required to see a minimum of two PAH-CHD or Fontan patients per month.

Patients were eligible for inclusion if they were aged over 18 years, had a clinician confirmed diagnosis of PAH-CHD or Fontan circulation associated with elevated PVR, and were not involved in a clinical trial at the time of the survey.

### Data collection

Clinicians completed online patient record forms (PRFs) for the next five consulting patients who visited the clinician in-person for routine care. PRFs captured patient demographics, disease characteristics, and treatment history. Clinicians completed PRFs through consultation of existing patient clinical records, as well as using their judgment and diagnostic skill, consistent with decisions made in routine clinical practice. The DSP is a cross-sectional survey, and no follow-up information was collected.

Patients whose clinicians completed a PRF were invited to complete a patient self-completion form (PSC) independently of their clinician to capture their own perspective on the impact and specificities of their condition. PRF and PSC data were matched anonymously using clinician/patient survey numbers.

### Measures and variables

All measures collected within the study are displayed in Table 1.

**Table 1:** Measures and Variables

Clinician/Patient reported	Measure	Timeframe of recall period
Clinician-reported	Clinician affiliation with a Pulmonary Hypertension Association accredited center	At survey date
	Clinician affiliation with an Adult Congenital Heart Association accredited center	At survey date
	CHD type	At survey date
	Patient age	At survey date
	Patient assigned sex at birth	At survey date
	Patient smoking status	At survey date
	Patient ethnicity	At survey date
	Patient concomitant conditions	At survey date
	Patient employment status	At survey date
	Patient unemployment/long term sick leave because of PAH-CHD	At survey date
	Current New York Heart Association Functional Classification	At survey date
	Age at CHD diagnosis	At CHD diagnosis
	Recorded gap in patient’s CHD management (above 1 year)	From CHD diagnosis to survey date
	Recorded gap in patient’s CHD management during the transition from pediatric to adult CHD care (above 1 year)	From CHD diagnosis to survey date
	Reasons for gap in CHD management	From CHD diagnosis to survey date
	Patient symptoms prior to diagnosis	Prior to PAH-CHD diagnosis
	Conditions investigated/suspected prior to PAH-CHD diagnosis	Prior to PAH-CHD diagnosis
	PAH-CHD diagnosing clinician	At PAH-CHD diagnosis
	Tests performed at PAH-CHD diagnosis	At PAH-CHD diagnosis
	Patient time to diagnosis (initial symptom presentation to correct diagnosis)	At PAH-CHD diagnosis
	Number of patient visits to a clinician for their PAH in the past 12 months	12 months prior to survey date
	Patient PAH-CHD symptoms at survey date	At survey date
	Patient PAH-CHD treatment at survey date	At survey date
	Reported barriers to PAH treatment	From PAH-CHD diagnosis
	Clinician feelings of prescribing restriction in general PAH-CHD management	At survey date
	Clinician reported tools / assessments used to determine patient suitability to receive treatment	At survey date
Patient-reported	Reported gap in management for PAH-CHD (over 1 year)	From PAH-CHD diagnosis to survey date
	Reasons for reported gap in PAH-CHD management	From PAH-CHD diagnosis to survey date
	First consulting clinician for PAH symptoms	Prior to PAH-CHD diagnosis
	Number of clinicians consulted prior to PAH-CHD diagnosis	Prior to PAH-CHD diagnosis
	Em-PHasis-10 scores	At survey date
	EQ-5D-5L VAS scores	At survey date
	EQ-5D-5L Index score	At survey date

Abbreviations: CHD, congenital heart disease; PAH, pulmonary arterial hypertension; EQ-5D-5L, EuroQol-5 Dimensions-5 Levels.

### Statistical analysis

Analyses were descriptive. Means are presented with standard deviation (SD), and data are split according to key subgroups of interest: consulting clinician affiliation with a Pulmonary Hypertension Association (PHA)-accredited center (affiliated/not affiliated); CHD type (PAH-CHD/Fontan patients); and clinician-perceived New York Heart Association functional class (NYHA-FC I-IV) at survey. Due to low patient numbers, NYHA-FC II and IV were grouped for analysis.

### Ethical and regulatory considerations

The survey was submitted to the Western Institutional Review Board, where ethical exemption determination was granted on 11/16/21 (Western Institutional Review Board work order (#21-ADRW-125). Patients provided informed consent in the PSC for use of their anonymized and aggregated data for research and publication in scientific journals. Data were collected so patients and clinicians could not be identified directly.

## Results

### Study population and clinical characteristics

Overall, 51 clinicians completed forms for 191 patients (98 PAH-CHD, 93 Fontan), of whom, 58 voluntarily completed PSCs (39 PAH-CHD, 19 Fontan patients; Table 2). A total of 30 clinicians (58.8%) reported an affiliation with a Pulmonary Hypertension Association (PHA) accredited center, 34 (66.7%) were affiliated with an Adult Congenital Heart Association accredited center, and 27 (52.9%) were affiliated with both. At the survey, a total of 65 patients were NYHA-FC I, 110 were NYHA-FC II and 16 were NYHA-FC III/IV.

**Table 2:** Study Sample

	Clinician survey sample	Patient record form sample	Patient self-complete sample
Adult cardiologist, *n* (%)	31 (60.8)	132 (69.1)	38 (65.5)
Pediatric cardiologist, *n* (%)	6 (11.8)	12 (6.2)	0 (0)
Adult pulmonologist, *n* (%)	14 (27.5)	47 (24.6)	20 (34.5)
**Total sample, *n* (%)**	**51 (100)**	**191 (100)**	**58 (100)**

*n* = population size.

Patient demographic data are summarized in Table 3. Of 191 patients, 62.3% were male and 70.7% were White/Caucasian, mean (SD) age was 38.8 years (15.9). Mean (SD) age of patients consulting a clinician affiliated with a PHA-accredited center were 37.7 (14.8) years, and for those not affiliated 41.3 (18.3) and the percentage of male patients was 67.4% and 50.0%, respectively. Mean (SD) age of Fontan patients and PAH-CHD patients was 35.8 (13.7) years and 41.6 (17.4) years, respectively, and percentage of male patients was 65.6% and 59.2%, respectively.

**Table 3:** Patient demographics.

		**Consulting clinician affiliation with PHA accredited center**		**Patient type**		**NYHA FC I–IV**		
	**Total**	**Affiliated**	**Not affiliated**	**PAH-CHD**	**Fontan patients**	**NYHA FC I**	**NYHA FC II**	**NYHA FC III/IV**

**Age**^a^								
n*	190	135	55	97	93	65	109	16
Mean (SD)	38.8 (15.9)	37.7 (14.8)	41.3 (18.3)	41.6 (17.4)	35.8 (13.7)	36.9 (12.8)	39.0 (16.8)	44.6 (20.8)
**Assigned sex at birth**								
n (%)	191	135	56	98	93	65	110	16
Male	119 (62.3)	91 (67.4)	28 (50.0)	58 (59.2)	61 (65.6)	44 (67.7)	6 (59.1)	10 (62.5)
Female	71 (37.2)	43 (31.9)	28 (50.0)	39 (39.8)	32 (34.4)	21 (32.3)	44 (40.0)	6 (37.5)
Intersex	1 (0.5)	1 (0.7)	0 (0)	1 (1.0)	0 (0)	0 (0)	1 (0.9)	0 (0)
**Ethnicity (≥5%)**								
White/Caucasian	135 (70.7)	98 (72.6)	37 (66.1)	63 (64.3)	72 (77.4)	45 (69.2)	78 (70.9)	12 (75.0)
African American	17 (8.9)	13 (9.6)	4 (7.1)	12 (12.2)	5 (5.4)	0 (0)	15 (13.6)	2 (12.5)
Hispanic /Latino	16 (8.4)	12 (8.9)	4 (7.1)	11 (11.2)	5 (5.4)	7 (10.8)	8 (7.3)	1 (6.2)
Other**	23 (12.1)	12 (8.9)	11 (19.7)	12 (12.1)	11 (12.0)	13 (19.9)	9 (8.1)	1 (6.2)
**Smoking status**								
Never smoked	118 (61.8)	89 (65.9)	29 (51.8)	53 (54.1)	65 (69.9)	40 (61.5)	71 (64.5)	7 (43.8)
Ex-smoker	42 (22.0)	25 (18.5)	17 (30.4)	30 (30.6)	12 (12.9)	13 (20.0)	25 (22.7)	4 (25.0)
Current smoker	7 (3.7)	6 (4.4)	1 (1.8)	4 (4.1)	3 (3.2)	0 (0)	3 (2.7)	4 (25.0)
Don’t know	24 (12.6)	15 (11.1)	9 (16.1)	11 (11.2)	13 (14.0)	12 (18.5)	11 (10.0)	1 (6.2)
**Employment status**								
Working full time	62 (32.5)	43 (31.9)	19 (33.9)	40 (40.8)	22 (23.7)	24 (36.9)	34 (30.9)	4 (25.0)
Working part time	43 (22.5)	28 (20.7)	15 (26.8)	17 (17.3)	26 (28.0)	20 (30.8)	21 (19.1)	2 (12.5)
Unemployed	29(15.2)	25 (18.5)	4 (7.1)	11 (11.2)	18 (19.4)	7 (10.8)	19 (17.3)	3 (18.8)
Student	25 (13.1)	20 (14.8)	5 (8.9)	8 (8.2)	17 (18.3)	10 (15.4)	14 (12.7)	1 (6.2)
Homemaker	14 (7.3)	8 (5.9)	6 (10.7)	9 (9.2)	5 (5.4)	3 (4.6)	11 (10.0)	0 (0)
Retired	14 (7.3)	8 (5.9)	6 (10.7)	12 (12.2)	2 (2.2)	0 (0)	11 (10.0)	3 (18.8)
On long-term sick leave	4 (2.1)	3 (2.2)	1 (1.8)	1 (1.0)	3 (3.2)	1 (1.5)	0 (0)	3 (18.8)
**Unemployed / on long-term sick leave as a result of their PAH-CHD?**								
n (%)	47	36	11	24	23	8	30	9
No	22 (46.8)	15 (41.7)	7 (63.6)	15 (62.5)	7 (30.4)	3 (37.5)	13 (43.3)	6 (66.7)
Yes	10 (21.3)	10 (27.8)	0 (0)	4 (16.7)	6 (26.1)	0 (0)	8 (26.7)	2 (22.2)
Don't know	15 (31.9)	11 (30.6)	4 (36.4)	5 (20.8)	10 (43.5)	5 (62.5)	9 (30.0)	1 (11.1)
**Current physician-perceived NYHA FC**								
Class I	65 (34.0)	39 (28.9)	26 (46.4)	29 (29.6)	26 (38.7)			
Class II	110 (57.6)	82 (60.7)	28 (50.0)	63 (64.3)	47 (50.5)			
Class III	13 (6.8)	11 (8.1)	2 (3.6)	6 (6.1)	7 (7.5)			
Class IV	3 (1.6)	3 (2.2)	0 (0)	0 (0)	3 (3.2)			
**CHD type**								
Fontan patient	93 (48.7)	71 (52.6)	22 (39.3)	0 (0)	93 (100.0)	36 (55.4)	47 (42.7)	10 (62.5)
PAH after corrective cardiac surgery	48 (25.1)	33 (24.4)	15 (26.8)	48 (49.0)	0 (0)	20 (30.8)	25 (22.7)	3 (18.8)
PAH associated with systemic-to-pulmonary shunts	26 (13.6)	17 (12.6)	9 (16.1)	26 (26.5)	0 (0)	5 (7.7)	21 (19.1)	0 (0)
PAH with small/coincidental defects	15 (7.9)	6 (4.4)	9 (16.1)	15 (15.3)	0 (0)	4 (6.2)	10 (9.1)	1 (6.2)
Eisenmenger syndrome	9 (4.7)	8 (5.9)	1 (1.8)	9 (9.2)	0 (0)	0 (0)	7 (6.4)	2 (12.5)
**Concomitant conditions (≥10%) top reported**								
None	66 (34.6)	38 (28.1)	28 (50.0)	29 (29.6)	37 (39.8)	33 (50.8)	30 (27.3)	3 (18.8)
Hypertension	37 (19.4)	29 (21.5)	8 (14.3)	26 (26.5)	11 (11.8)	11 (16.9)	22 (20.0)	4 (25.0)
Anxiety	28 (14.7)	21 (15.6)	7 (12.5)	16 (16.3)	12 (12.9)	6 (9.2)	19 (17.3)	3 (18.8)
Depression	24 (12.6)	19 (14.1)	5 (8.9)	15 (15.3)	9 (9.7)	3 (4.6)	15 (13.6)	6 (37.5)
Gastroesophageal reflux disease	21 (11.0)	19 (14.1)	2 (3.6)	11 (11.2)	10 (10.8)	3 (4.6)	14 (12.7)	4 (25.0)
Asthma	20 (10.5)	18 (13.3)	2 (3.6)	10 (10.2)	10 (10.8)	7 (10.8)	7 (6.4)	6 (37.5)
Elevated cholesterol/hyperlipidemia	20 (10.5)	18 (13.3)	2 (3.6)	13 (13.3)	7 (7.5)	3 (4.6)	13 (11.8)	4 (25.0)

Source: clinician-reported PRF data.

n = population size

CHD, congenital heart disease; FC, functional class; NYHA, New York Heart Association; PAH, pulmonary arterial hypertension; PRF, patient record form; SD, standard deviation; PHA, pulmonary hypertension association

* Reported base numbers are applicable for each subsection unless otherwise specified

** Includes Native American, Asian (Indian subcontinent), Asian (other), Middle Eastern, Mixed race, South-East Asian, other.

a n=190, with one patient reported to be 90+ and a mean age was not captured age

Most patients (65.4%) had at least one concomitant comorbidity, most frequently hypertension (19.4%), anxiety (14.7%), and depression (12.6%). The proportion of patients with a concomitant condition ranged from 49.2% for NYHA FC I to 81.2% for NYHA FC III/IV. Please see supplementary information (Appendix A, Table 2**;** Figure S1) for patient-reported demographics and quality of life datapoints.

### CHD history and transition from child to adult services

The majority of patients (72.8%) were diagnosed with CHD in childhood, with 17.3% of patients diagnosed after age 18. In patients who underwent Fontan palliation versus other forms of PAH-CHD, initial CHD diagnosis was more common at <5 months of age (36.6% vs 21.4%, respectively).

Clinicians reported 10.5% of patients (*n* = 20) had a recorded gap of ≥1 year in their CHD management, of which 80.0% of cases occurred during the transition period from pediatric to adult CHD care. Of those with a treatment gap, the most common clinician-reported reasons were that the patient was feeling well and felt follow-up was unnecessary (37.5%) or the patient being unaware of the lifelong care requirements of their disease (37.5%). A gap in care was reported for 6.5% of Fontan patients and 14.3% of patients with PAH-CHD.

Just under a third (29.3%) of patients self-reported going ≥1 year without seeing a clinician for management of their PAH-CHD. The most common reasons for this were geographic relocation (70.6%) and believing follow-up was unnecessary due to self-perceived good health (64.7%).

### PH diagnostic journey and testing

In the total sample, the most common clinician-reported symptoms present before diagnosis were exertional dyspnea (52.4%) and fatigue (39.8%)**.** Patients most often presented to their primary care physicians (56.4%) for their initial PAH symptoms. Patients consulted with an average of 2.0 (1.0) healthcare professionals before receiving a diagnosis of PAH-CHD or Fontan-associated increased PVR. An alternative diagnosis was initially suspected for 76.4% of patients, most commonly asthma (43.1%), anxiety (40.2%), and congestive heart failure (36.3%). A mean of 3.1 (2.6) conditions were investigated before confirmatory diagnosis. PAH diagnosis was most often confirmed by an adult treating cardiologist (57.6%) or adult treating pulmonologist (18.8%).

**Figure 1:**
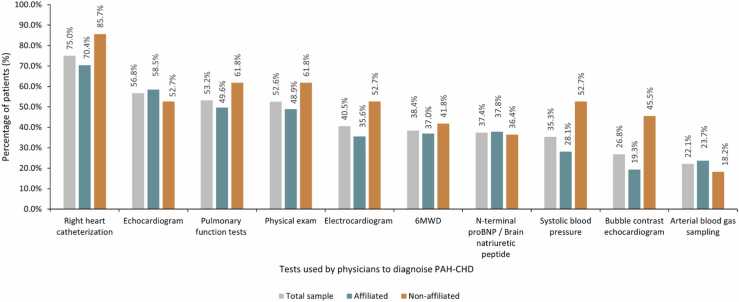
*Clinician-reported tests used to diagnose pulmonary arterial hypertension—congenital heart disease or confirm elevated pulmonary vascular resistance by total sample and clinician affiliation with a Pulmonary Hypertension Accredited center.* Clinician-reported PRF data. Affiliated is defined as the treating clinician being affiliated with a Pulmonary Hypertension Association (PHA) accredited Center; Non-affiliation is defined as the treating clinician not being affiliated with a PHA accredited center. 6MWD, Six-minute walking distance; BNP, Brain natriuretic peptide; PAH-CHD, Pulmonary arterial hypertension congenital heart disease; PH, Pulmonary hypertension; PRF, patient record form; PVR, Pulmonary vascular resistance.

Clinicians reported 75.0% of patients underwent right heart catheterization (RHC) to confirm diagnosis. This varied by consulting clinicians’ affiliation with a PHA accredited center (Figure 1). The most common reported reasons for forgoing RHC were patient refusal (40.0%) and perceived high patient risk (11.0%).

Clinicians reported a mean of 1.7 (2.2) years from initial symptom presentation to correct diagnosis/confirmation (*n* = 143). Mean reported time was 1.4 (2.0) years for Fontan patients and 2.0 (2.3) years for patients with PAH-CHD, and tended to be longer among NYHA FC III/IV patients (2.4 [2.0] years, *n* = 11). Clinician-reported mean number of visits in the last 12 months for PAH was 2.6 (*n* = 191).

### Current symptomatology

**Figure 2:**
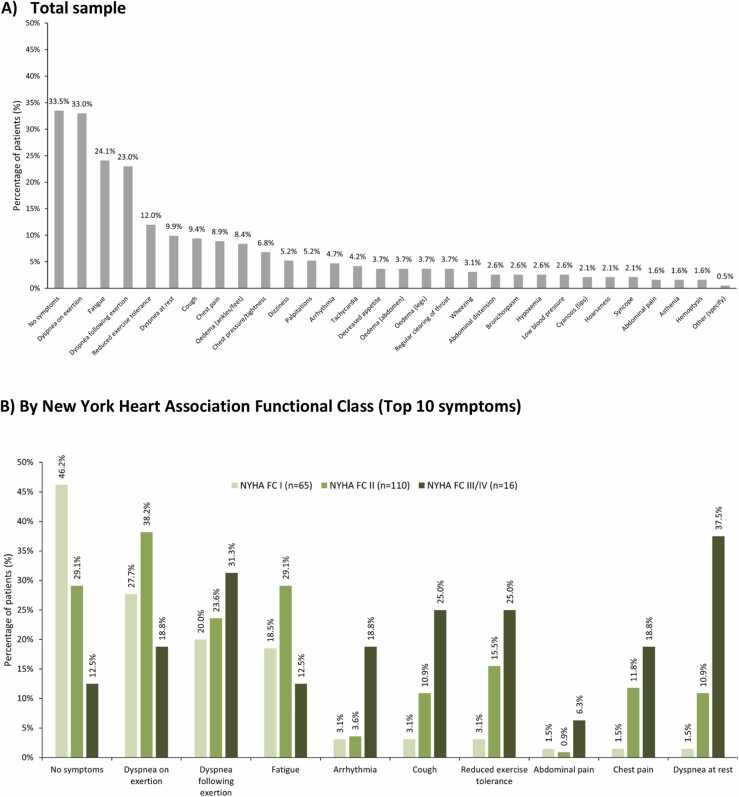
*Clinician-reported pulmonary arterial hypertension symptoms at survey date. **(A)** Total sample; **(B)** By New York Heart Association Functional Class.* Clinician-reported PRF data. NYHA FC, New York heart association functional class; PAH, Pulmonary arterial hypertension; PRF, patient record form. Symptoms refer to those the clinician attributed to PH, not CHD.

Clinicians reported that 66.5% of patients experienced symptoms in the 4 weeks prior to the survey date, including 62.4% of Fontan patients and 70.4% of PAH-CHD patients. Symptom reporting increased with greater NYHA FC (FC I, 53.8%; FC II, 70.9%; FC III/IV 87.5%). The most common clinician reported active symptoms were dyspnea during exertion (33.0%), fatigue (24.1%), and dyspnea following exertion (23.0%) (Figure 2). Patients reported symptoms of fatigue (57.1%), shortness of breath following exertion (53.6%), and shortness of breath during exertion (51.8%).

### Treatment

Clinicians reported that 75.9% of patients were prescribed treatment for their PAH-CHD or elevated PVR at the survey, most commonly chronic maintenance therapy (93.1%) versus a minor proportion receiving a treat and repair treatment strategy (6.9%). In total, 77.8% of patients received first-line maintenance treatment, with 17.0%, 4.4%, and 0.7% requiring a switch to second-, third- and fourth-line maintenance treatments, respectively. All NYHA FC I patients remained on first-line maintenance treatment compared with just 20.0% for NYHA FC III/IV (Figure S2).

**Figure 3:**
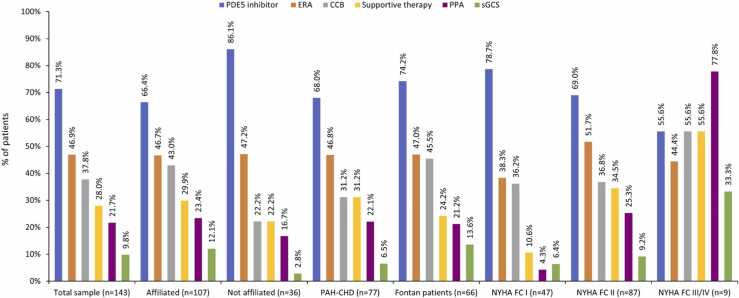
*Clinician-reported prescribed treatment class at the survey date (% of patients) by total sample, treating clinician affiliation with a Pulmonary Hypertension Accredited center, congenital heart disease type and by New York Heart Association Functional Class at the survey date.* Clinician-reported PRF data. Affiliated is defined as the treating clinician being affiliated with a Pulmonary Hypertension Association (PHA) accredited Center; Non-affiliation is defined as the treating clinician not being affiliated with a PHA accredited center. CCB, Calcium channel blocker; ERA, Endothelin receptor antagonist; PDE5, Phosphodiesterase type 5; PPA, Prostacyclin pathway agent; sGCS, Soluble guanylate cyclase stimulator; NYHA FC, New York Heart Association Functional Class; Supportive therapies includes any patients prescribed a diuretic, oxygen, calcium channel blocker, and other therapies.

Of treated patients, 84.7% of patients were prescribed a PH-specific therapy (defined as any of the following treatment classes: phosphodiesterase-5 [PDE5] inhibitors, endothelin receptor antagonists [ERAs], soluble guanylate cyclase stimulators [sGCSs], and prostacyclin pathway agents [PPAs]). This was prescribed as monotherapy for 37.1% of patients, with 47.6% prescribed combination therapy. In total, 15.4% of patients were prescribed supportive therapies alone (calcium channel blocker, diuretic, anticoagulant, or oxygen). PDE5 inhibitors were the most common PAH-specific treatment prescribed (71.3%), followed by ERAs (46.9%), PPAs (21.7%), and sGCSs (9.8%) (Figure 3). Patients receiving PDE5 inhibitors (*n* = 102) were treated with sildenafil (50.0%), tadalafil (48.0%), udenafil (4.9%), and avanafil (1.0%).

**Figure 4:**
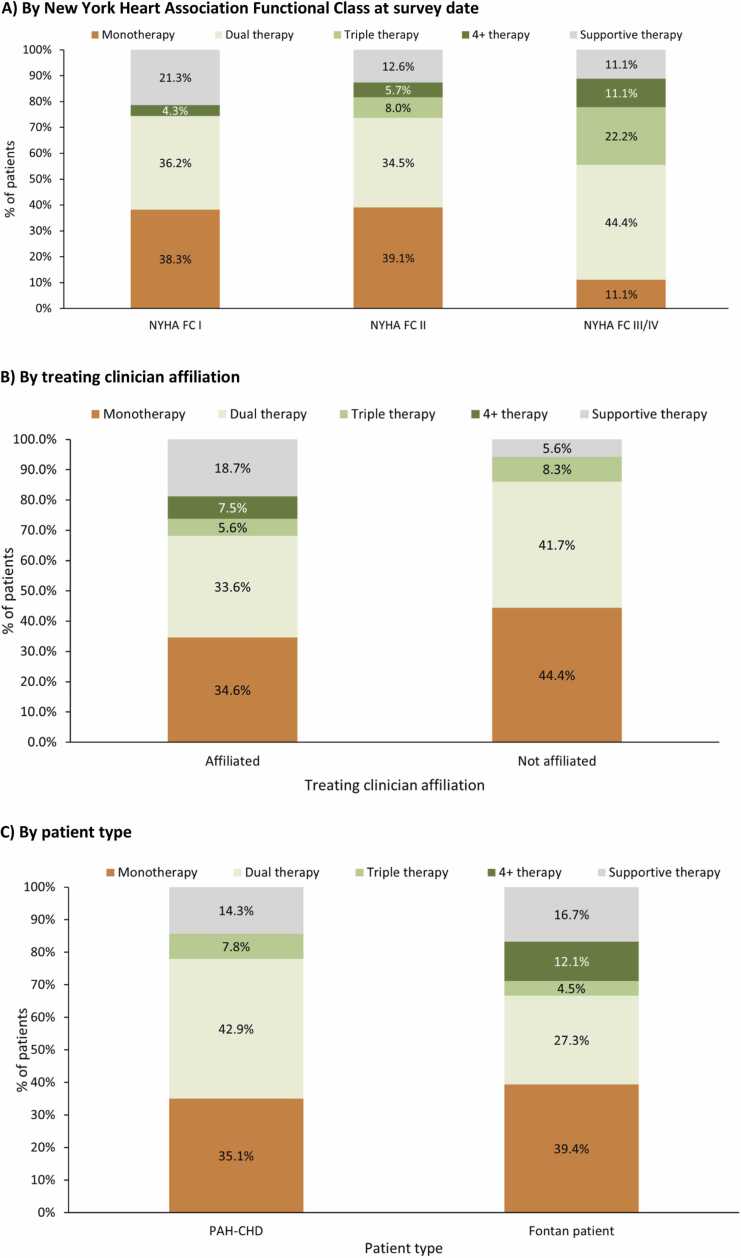
*Clinician-reported prescribed pulmonary hypertension treatment intensity at survey date by **(A)** New York Heart Association Functional Class; **(B)** Treating clinician affiliation with a Pulmonary Hypertension Association accredited center; and **(C)** Congenital heart disease type (% of patients).* Clinician-reported PRF data. Affiliated is defined as the treating clinician being affiliated with a Pulmonary Hypertension Association (PHA) accredited Center; Non-affiliation is defined as the treating clinician not being affiliated with a PHA accredited center. CHD, Congenital heart disease; NYHA FC, New York Heart Association Functional Class; PAH, Pulmonary arterial hypertension; PH, Pulmonary hypertension. Monotherapy, includes any patients prescribed one PAH specific therapy (defined as ERAs, PDE5 inhibitors, sGCSs, and PPAs), Dual therapy, includes any patients prescribed two PAH specific therapies, Triple therapy, includes any patients prescribed three PAH specific therapies, 4+therapies, includes any patients prescribed 4 PAH specific therapies, supportive therapies includes any patients prescribed a diuretic, oxygen, calcium channel blocker, and other therapies.

Patients on monotherapy were most commonly prescribed PDE5 inhibitors (75.5%), and PDE5 inhibitors with ERAs were the most frequent form of combination therapy (82.4%). Usage of PH-specific combination therapy split by NYHA-FC is shown in Figure 4A. PPAs were prescribed to 77.8% of FC III/IV patients, 25.3% of FC II patients, and 4.3% of FC I patients. Combination therapy usage was similar irrespective of the treating clinician's affiliation with a PHA-accredited center (Figure 4B). The proportion of Fontan patients prescribed combination therapy was lower than in PAH-CHD patients (Figure 4C).

One-third of patients experienced barriers to PAH treatment during their condition management, with a higher proportion of Fontan patients reported to have had issues accessing treatment (51.4% vs 16.2% PAH-CHD). Common barriers included cost (45.8%), lack of insurance coverage (22.9%), and patient noncompliance (20.8%).

### Clinician perception

**Figure 5:**
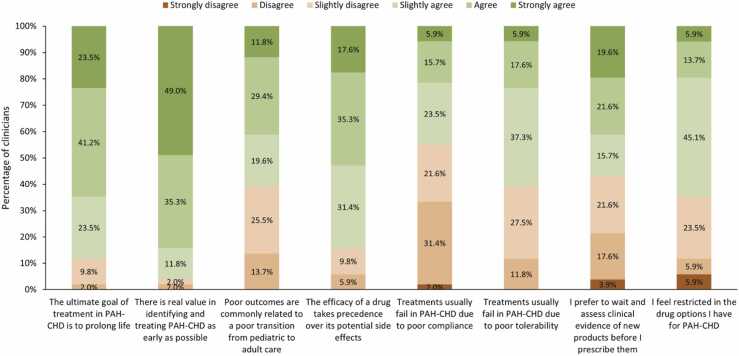
*Clinician attitudes.* Clinician-reported survey data. CHD, Congenital heart disease; PAH, Pulmonary arterial hypertension.

In total, 64.7% of clinicians agreed (slightly agree, agree, or strongly agree) that they felt restricted in the treatments they could prescribe (64.7%; Figure 5). Clinicians (*n* = 51) reported using symptom severity (86.3%) to determine patient suitability to receive treatment. Only 9.9% of clinicians reported utilizing PAH risk assessment tools to determine patient’s suitability to receive treatment, with 5.9% reporting utilizing the REVEAL 2.0, 2.0% utilizing the REVEAL 2.0 L and 2.0% utilizing other risk assessment measures.

## Discussion

There is limited real-world evidence surrounding the treatment and management of PAH and elevated PVR in patients with CHD throughout adulthood, including during the period of transition from pediatric care. Our study adds to the growing body of evidence from the international COMPERA-CHD registry regarding demographics, clinical characteristics, treatment patterns, and outcomes in PAH-CHD and Fontan patients.^15^, ^18^ We have also gained insights into the real-world management of these patients, including patterns of adherence to guideline directed care Lastly, our data highlights the substantial symptom burden and quality of life impact seen in this population.

Demographic characteristics of the current sample differ slightly from demographic reports captured in COMPERA. In our cohort, most patients were male, and the mean (SD) age was 38.8 (15.9), compared to reports captured in COMPERA, where the mean age was 45.3 (16.8) years and 33.4% were male. This could be due to the high proportion of Fontan patients—making up nearly half of our sample—who tend to be younger than other PAH-CHD subgroups, compared to only 9% in COMPERA.^18^

The ability to ensure continuous care and successfully transition CHD patients from pediatric to adult care continues to lag behind the needs of the growing adult CHD population. A study by Hilderson et al^25^ found that 74% of US and European pediatric cardiology programs transferred pediatric CHD patients to adult-focused care, but only a third of centers provided a structured transition program for patients. A further study by Gurvitz et al (2013) found nearly half of CHD patients experienced more than a 3-year gap in the course of their cardiology care, most commonly first occurring at around 19 years.^26^ To our knowledge this is the first real-world survey assessing this gap from transition to adult care specifically among CHD patients with pulmonary vascular disease.

Our data suggests that the frequency in which gaps in care occur may be underrecognized by clinicians, given the discrepancy between clinician-reported gaps in 10% of patients versus a third of our patients self-reporting gaps in care. The implications of this are underscored by the fact that our patient population with pulmonary vascular disease (on therapy in the majority of cases) are among the highest risk CHD group in terms of cardiovascular morbidity and mortality.^6^ While the importance of patient education and a structured transition process from pediatric to adult CHD care has been increasingly recognized, we may not have moved the needle much over the past decade in ensuring continuity of care. Future research should look to explore the underlying causes of the disconnect between physician-reported and patient-reported gaps in care.

Patients frequently reported experiencing symptoms. Consistent with other studies in PAH,^27^ dyspnea on exertion and fatigue were most commonly reported, with the highest symptom burden reported in the NYHA FC III/IV group. Interestingly, a third of patients labeled as FC I also reported experiencing dyspnea, indicating symptoms may be underrecognized in this complex group who often have baseline exertional limitations. Despite pulmonary vascular disease being a known complication of CHD, alternative diagnoses were suspected in most symptomatic patients, and PAH/elevated-PVR diagnosis was significantly delayed, even in Fontan patients. We found patients waited an average of 1.7 years for diagnosis, with initial symptoms often being misattributed to other respiratory conditions or anxiety. Delays to diagnosis and treatment in patients with PH are associated with a detrimental impact to patient survival and quality of life.^10^, ^28^ High rates of misdiagnosis and diagnostic delays in PAH-CHD have been reported for many years, highlighting the need for further education around the associated complications of PH.^29^, ^30^, ^31^

The insights from this study into current treatment of PAH-CHD patients are valuable in our understanding of this rare patient segment and can contribute to the growing body of evidence. Most patients were receiving targeted PAH treatment, with roughly half (47.6%) receiving combination therapy, with differences reported across disease types and settings. The relatively lower use of combination therapies in the Fontan sample is consistent with findings from the COMPERA registry.^18^ While the reason for lower usage was not explored, this could reflect previously reported barriers to PAH prescribing or a lack of consensus of the role of PAH therapies in the Fontan population.^10^, ^32^ Limited use of combination therapies in PAH-CHD has been seen in other studies. In the COMPERA registry, treatment at inclusion was predominantly monotherapy (69%), although this shifted to favor combination therapy (53%) in patients who had at least one follow-up visit.^18^ Lower use of combination therapy was found in the Spanish REHAP National Registry up to May 2013, where just 9.5% of PAH-CHD patients were initially started with combination therapy, from 1.3% of small shunt patients to 10.9% of postoperative patients.^33^ The available information on currently available treatments from clinical trials is relatively limited, with trials such as GRIPHON,^34^ SERAPHIN,^35^ and PATENT^36^ including a limited PAH-CHD patient sample (110, 62, and 35, respectively). There is therefore a need to better understand treatment patterns and explore the benefits of combination therapy in the CHD population. It is vital to research the applicability of treatment guidelines in PAH-CHD and Fontan patients.

### Limitations

Several limitations should be considered when interpreting of our findings.

Participating patients may not reflect the general PAH-CHD population since the DSP only includes patients who consulted with their clinician. The DSP is based on a pseudo-random sample of clinicians or patients. While minimal inclusion criteria governed the selection of participating clinicians, participation was influenced by their willingness to complete the survey. The DSP also only includes patients within the US and so only reflects PAH-CHD management in the US.

To minimize selection bias, clinicians were asked to provide data for a consecutive series of patients. Patient eligibility was based on the judgment of respondent clinicians and not a formalized diagnostic checklist. There was no requirement for RHC to confirm diagnosis. While this approach reflects clinician’s real-world classification of their patients, it also introduces the potential for misdiagnosis.

## Conclusions

This US-based real-world study was able to investigate the journey from initial CHD diagnosis to PH management and assessed gaps during transition from pediatric to adult care for a patient group not well characterized in existing literature. Despite being an at risk population who are typically closely monitored from early childhood onward, CHD patients experience similar delays between symptom onset and diagnosis/confirmation as seen in other PAH etiologies. This study also reveals gaps in the CHD management paradigm, especially when transitioning from pediatric to adult CHD care. Despite a diagnosis of PAH-CHD, we observed trends for low utilization of diagnostic tests such as RHC and low combination therapy usage. Few clinicians reported using risk assessment tools in PAH-CHD management, highlighting an area for targeted education. This study highlights the potential for future research in the transition of care from pediatric to adult specialty CHD care, delay of PAH-CHD diagnosis, testing for PH, and examination of real-world treatment patterns.

## CRediT authorship contribution statement

Jordan D. Awerbach: Writing - review and editing. Carly J Paoli: Writing - review and editing. Megan Scott: Writing - original draft and Writing - review and editing. Gurinderpal Doad: Writing - review and editing. Julia Harley: Data curation, Investigation, Resources, Visualization, Writing - original draft and Writing - review and editing. Daniel Graham: Data curation, Investigation, Resources, Visualization, Writing - original draft and Writing - review and editing. Mark Small: Project administration, Supervision, Writing - original draft and Writing - review and editing. Sumeet Panjabi: Writing - review and editing. Leigh C. Reardon: Writing - review and editing.

## Financial support

Johnson & Johnson Innovative Medicine did not influence the original survey through either contribution to the design of questionnaires or data collection. The analysis described here used data from the Adelphi Real World PAH-CHD DSP. The DSP is a wholly owned Adelphi Real World product. Johnson & Johnson Innovative Medicine is one of multiple subscribers to the DSP. Publication of survey results was not contingent on the subscriber's approval or censorship of the publication.

## Declaration of Competing Interest

The authors declare the following financial interests/personal relationships, which may be considered as potential competing interests: C. J. Paoli, S. Panjabi, and G. Doad are employees of Actelion Pharmaceuticals US, Inc., a Johnson & Johnson company. J. Harley, D. Graham, M. Scott, and M. Small are full-time employees of Adelphi Real World. L. Reardon and J. Awerbach received consulting fees from Johnson & Johnson Innovative Medicine.

## Glossary of Abbreviations

CCB: Calcium Channel Blocker
CHD: Congenital Heart Disease
DSP: Disease Specific Programme
ERA: Endothelin Receptor Antagonist
HRQoL: Health-Related Quality of Life
NYHA FC: New York Heart Association Functional Class
PAH: Pulmonary Arterial Hypertension
PAH-CHD: Pulmonary Arterial Hypertension - Congenital Heart Disease
PDE5: Phosphodiesterase-type 5
PH: Pulmonary Hypertension
PPA: Prostacyclin pathway agents
PRF: Patient Record Form
PSC: Patient Self-Completion
PVR: Pulmonary Vascular Resistance
RHC: Right Heart Catheterization
SD: Standard Deviation
sGCS: Soluble Guanylate Cyclase Stimulator
US: United States
VAS: Visual Analogue Scale
WIRB: Western Institutional Review Board
6MWD: 6 Minute walking Distance

## Footnotes

Appendix A Supplementary data associated with this article can be found in the online version at doi:10.1016/j.jhlto.2025.100326.
