# Supplementary material for: Real-world experiences and treatment patterns among congenital heart disease patients with associated pulmonary vascular disease: Results from a real-world survey in the United States
Source: JHLT Open. 2025 Jun 20;9:100326. doi: 10.1016/j.jhlto.2025.100326 (PMC12343480; doi:10.1016/j.jhlto.2025.100326)
Supplement: Supplementary file 1 — Supplementary material [file mmc1.docx]

**Figure S1. emPHasis-10 scores for each of the 10 items (total population, % of patients)**

Patient-reported data

emPHasis-10 domains are scored as follows: Breathlessness (0, I am not frustrated by my breathless; 5, I am very frustrated by my breathlessness); Breathlessness impact (0, being breathlessness never interrupts my conversations; 5, being breathlessness always interrupts my conversations); Resting (0, I do not need to rest during the day; 5, I always need to rest during the day); Exhausted (0, I do not feel exhausted; 5, I always feel exhausted); Energy (0, I have lots of energy; 5, I have no energy at all); Stairs (0, when I walk up one flight of stairs I am not breathless; 5, when I walk up one flight of stairs I am very breathless); Confidence (0, I am confident out in public places/crowds despite my PH; 5, I am not confident out in public places/crowds because of my PH); Control (0, PH does not control my life; 5, PH completely controls my life); Independence (0, I am independent; 5, I am completely dependent); Burden (0, I never feel like a burden; 5, I always feel like a burden)

**Figure S2. Clinician-reported prescribed treatment class at survey date by line of therapy (% of patients)**

Clinician-reported PRF data

Supportive therapies, defined as any of calcium channel blocker, diuretic, anticoagulant, or oxygen; PDE5 inhibitor, phosphodiesterase-5 inhibitors; ERA, Endothelin receptor antagonist; PPA, Prostacyclin pathway agents; SGCSs, Soluble guanylate cyclase stimulators

**Table S1. Physician-reported patient demographic data by patient self-completion status**

|  | **PSC completed** | **PSC not completed** |
| --- | --- | --- |
|  |  |  |
| **Physician-reported age (years) at survey data, n** | **58** | **132** |
| Mean | 38.0 | 39.1 |
| Min | 18 | 18 |
| Max | 85 | 89 |
| SD | 17.33 | 15.32 |
| **Physician-reported patient sex, n (%)** | **58** | **133** |
| Male | 39 (67.3%) | 80 (60.2%) |
| Female | 18 (31%) | 53 (39.8%) |
| Intersex | 1 (1.7%) | 0 (0%) |
| **Physician-reported BMI, n** | **58** | **133** |
| Mean | 26.3 | 25.6 |
| Min | 18 | 17 |
| Max | 40 | 45 |
| SD | 4.69 | 4.22 |
| **Physician-reported patient ethnicity, n (%)** | **58** | **133** |
| White/Caucasian | 46 (79.3%) | 89 (66.9%) |
| African American | 7 (12.1%) | 10 (7.5%) |
| Native American | 0 (0%) | 3 (2.2%) |
| Asian (Indian subcontinent) | 0 (0%) | 3 (2.3%) |
| Asian (other) | 0 (0%) | 3 (2.3%) |
| Hispanic / Latino | 3 (5.2%) | 13 (9.8%) |
| Middle Eastern | 1 (1.7%) | 2 (1.5%) |
| Mixed race | 0 (0%) | 9 (6.8%) |
| South-East Asian | 1 (1.7%) | 1 (0.7%) |
| Other | 0 (0%) | 0 (0%) |
| **Physician-reported patient smoking status, n (%)** | **58** | **133** |
| Current smoker | 3 (5.2%) | 4 (3%) |
| Ex-smoker | 16 (27.6%) | 26 (19.6%) |
| Never smoked | 37 (63.8%) | 81 (60.9%) |
| Don’t know | 2 (3.4%) | 22 (16.5%) |
| **Physician-reported patient employment status, n (%)** | **58** | **133** |
| Working full time | 21 (36.2%) | 41 (30.8%) |
| Working part time | 14 (24.1%) | 29 (21.8%) |
| On long term sick leave | 0 (0%) | 4 (3%) |
| Homemaker | 3 (5.2%) | 11 (8.3%) |
| Student | 8 (13.8%) | 17 (12.8%) |
| Retired | 8 (13.8%) | 6 (4.5%) |
| Unemployed | 4 (6.9%) | 25 (18.8%) |
| Furloughed / Government work scheme | 0 (0%) | 0 (0%) |
| **Physician-reported patient < unemployed / on long term sick leave / retired > as a result of their PAH-CHD, n (%)** | **12** | **35** |
| Yes | 3 (25%) | 7 (20%) |
| No | 8 (66.7%) | 14 (40%) |
| Don't know | 1 (8.3%) | 14 (40%) |
| **Physician-reported patient PAH-CHD type, n (%)** | **58** | **133** |
| Eisenmenger syndrome | 5 (8.6%) | 4 (3%) |
| PAH associated with systemic-to-pulmonary shunts | 9 (15.5%) | 17 (12.8%) |
| PAH with small/coincidental defects | 1 (1.7%) | 14 (10.5%) |
| PAH after corrective cardiac surgery | 24 (41.4%) | 24 (18.1%) |
| Fontan patient | 19 (32.8%) | 74 (55.6%) |

Clinician-reported PRF data.

PSC, Patient self-completion; PAH, Pulmonary arterial hypertension; CHD, Congenital heart disease.

n = population size

**Table S2. Mean EQ-5D-Visual Analogue Score, EQ-5D-5L and emPHasis-10 scores (Total sample, clinician affiliation with a Pulmonary Hypertension Accredited center, Patient type and current New York Heart Association Functional Class, % population)**

|  |  | **Treating clinician affiliation with PHA accredited center** | | **Patient type** | | **NYHA FC I–IV** | | |
| --- | --- | --- | --- | --- | --- | --- | --- | --- |
|  | **Consecutive sample** | **Affiliated** | **Not affiliated** | **PAH-CHD** | **Fontan patients** | **NYHA FC I** | **NYHA FC II** | **NYHA FC III/IV** |
|  |  |  |  |  |  |  |  |  |
| **EQ-5D-5L VAS score** |  |  |  |  |  |  |  |  |
| n | 56 | 51 | 5 | 37 | 19 | 16 | 36 | 4 |
| Mean | 70.0 | 69.4 | 76.0 | 66.8 | 76.3 | 80.5 | 66.9 | 56.0 |
| Min | 20.0 | 20.0 | 60.0 | 20.0 | 20.0 | 70.0 | 20.0 | 20.0 |
| Max | 100.0 | 100.0 | 95.0 | 100.0 | 95.0 | 95.0 | 100.0 | 84.0 |
| SD | 22.5 | 23.2 | 12.9 | 23.4 | 19.6 | 8.3 | 24.5 | 31.2 |
| **EQ-5D-5L Index score** |  |  |  |  |  |  |  |  |
| n | 55 | 50 | 5 | 36 | 19 | 16 | 55 | 4 |
| Mean | 0.7 | 0.7 | 0.8 | 0.7 | 0.8 | 0.9 | 0.7 | 0.5 |
| Min | 0 | 0 | 1 | 0 | 0 | 1 | 0 | 0 |
| Max | 1.0 | 1.0 | 1.0 | 1.0 | 1.0 | 1.0 | 1.0 | 1.0 |
| SD | 0.21 | 0.21 | 0.15 | 0.22 | 0.17 | 0.13 | 0.19 | 0.26 |
| **emPHasis-10 score** |  |  |  |  |  |  |  |  |
| n | 56 | 51 | 5 | 37 | 19 | 16 | 36 | 4 |
| Mean | 26.4 | 27.2 | 18.4 | 27.2 | 24.8 | 15.4 | 30.1 | 37.0 |
| Min | 0 | 0 | 6 | 2 | 0 | 0 | 2 | 28 |
| Max | 50 | 50 | 26 | 50 | 48 | 29 | 50 | 44 |
| SD | 11.72 | 11.78 | 8.02 | 11.83 | 11.64 | 8.11 | 10.08 | 6.83 |

CHD, Congenital heart disease; NYHA FC, New York heart association functional class; VAS, Visual analogue scale

n = population size

Patient HRQoL was assessed via the five-level version of the EQ-5D and the emPHasis-10. EQ-5D index scores were calculated according to US-specific tariffs, in which scores begin at 1 (indicating a full health state) and are negatively scored to 0 (indicating a health state equivalent to dead). Via the EQ-VAS, patients provided a score of their current health on a scale from 0 (‘The worst health you can imagine’) to 100 (‘The best health you can imagine’). emPHasis-10 is a PH-specific tool based on a questionnaire consisting of 10 items formatted as a semantic six-point differential scale resulting in patient-reported scores ranging from 0 to 50. The maximum total score for emPHasis-10 is 50, where a higher score represents a higher symptom burden.

Affiliated is defined as the treating clinician being affiliated with a Pulmonary Hypertension Association (PHA) accredited Centre; Non-affiliation is defined as the treating clinician not being affiliated with a PHA accredited center
